# Neurobehavioral Effects of Low-Dose Chronic Exposure to Insecticides: A Review

**DOI:** 10.3390/toxics11020192

**Published:** 2023-02-19

**Authors:** Laura Maria Antonangeli, Saniya Kenzhebekova, Claudio Colosio

**Affiliations:** 1 Post Graduate School in Occupational Medicine, University of Milan, 20122 Milano, Italy; 2 Department of Health Sciences, University of Milan, International Centre for Rural Health of the Santi Paolo e Carlo ASST of Milan, 20142 Milano, Italy

**Keywords:** insecticides, neurobehavioral effects, neurobehavioral testing, occupational exposure

## Abstract

The modes of action of insecticides frequently involve a neurotoxic effect; therefore, the study of neurotoxic effects caused by long-term and low-dose insecticide exposure is of particular interest. This study looks at whether or not new studies conducted after 2009 and up to 2021 have provided new evidence for a better understanding of the actual neurobehavioral risk associated with long-term insecticide exposure. We selected and reviewed studies carried out on the neurobehavioral effects of neurotoxic insecticides (organophosphates and/or carbamates, pyrethroids, multiple or undefined insecticides, and organochlorines) considering occupational and non-occupational exposures. The articles were also scored and ranked based on seven parameters. Eighty-six studies were chosen for a final review process from among the 950 scientific papers identified. Twenty-six addressed occupational exposure and six environmental exposure. Among the latter group of studies, 17 focused on rural residents, to be assumed exposed because of living in rural areas, and 43 on the general population. Pending doubts have not been resolved in the last ten years due to the presence of contradictory and hardly comparable results and the fact that in most of the studies showing an evident neurobehavioral impairment the frequent presence of a previous episode of poisoning and hospitalization, with severe brain hypoxia, impaired the possibility of confirming the presence of a causal association with insecticide exposure. Interestingly, the most severely exposed groups, such as applicators who did not wear personal protective equipment, performed worse on neurobehavioral tests. As for residential exposure, there is sufficient evidence to suggest that prenatal OP exposure may increase the risk of ADHD in children.

## 1. Introduction

Because the modes of action of most insecticides are based on neurotoxicity, the study of neurotoxic effects caused by long-term and low-dose insecticide exposure is of particular interest. Among neurotoxic insecticides, we can list carbamates, formamidine, morpholine derivatives, neonicotinoids, organophosphorus compounds, organochlorinated compounds, and pyrethroids. The modes of action of neurotoxicity are diverse, but all these compounds, due to their neurotoxic properties, may bring about an impairment of different physiological functions in long-term exposure conditions [1,2,3,4]. Among them, an area of particular interest is represented by behavior.

Human behavior is based on different functions of the nervous system [5]. It relies both on the integrity of the physiological substrate and species–specific mechanisms at the level of the individual and inter-individual interactions. At the level of fundamental biochemistry and physiology, brain energetics and neurotransmission affect development, learning, emotion, and behavior [6]. Behavior can mainly be considered a combination of four parameters: cognitive, sensory-motor, psychological, and psychomotor. The bellwether of the damage to the nervous system is the changes in behavior, which is why behavioral measures are valid indicators of nervous system function. Behavioral changes can occur even before the detection of brain alterations, making behavior evaluation particularly appropriate for risk assessment [7,8].

The assessment of neurobehavioral impairment needs a complex approach, based on the outcome of different complementary tests, each addressing specific brain functions (Table 1). Different research groups adopt different batteries of neurobehavioral tests for their research, making comparisons between different studies very difficult. In the best study conditions, all four parameters (cognitive, sensory-motor, psychological, and psychomotor) can be studied within the study time frame. For neurobehavior testing, the World Health Organization has recommended, since 1983, the “Neurobehavioral Core Test Battery (NCTB)”, which is the international standard for diagnosing unfavorable human behavioral consequences related to neurotoxic chemical exposure. However, as guidance to the study of human neurotoxicology, a screening battery of more broadly sensitive tests that represent the fields of neuropsychology, experimental psychology, and neurology can be added [9].

In 2003 and 2009, respectively, our group published two contributions on this matter [10,11]. The main findings have been that comparison among different studies is difficult because of significant differences among study protocols and the parameters measured. Some studies report a reduction of performance in all functions [12,13], others demonstrate performance decrements only in specific parameters but not in others [14], and some studies are fully negative [15,16]. Not everyone reacts the same; gene variations may produce differences in serotonergic or dopaminergic responses or reactions, and that may mitigate the impact of environmental factors on a variety of psychological consequences. This indicates that people may be more or less sensitive to environmental conditions depending on their genotype [17]. Our main conclusions were that overt neurotoxicological effects occurred only as sequelae of acute intoxication, while there was no solid evidence of neurobehavioral effects as a consequence of long-term exposure to low doses of pesticides, even though in very high exposure conditions some slight effects have been observed, especially in cognitive functions.

This study aims to assess whether, after 2009 and up to 2021, new studies have brought new evidence for a better understanding of the actual neurobehavioral risk in long-term insecticide exposure.

## 2. Materials and Methods

### 2.1. Literature Search

A broad review of the articles published from May 2009 until September 2021 was conducted in Medline/PubMed, Scopus, and Web of Science using the following search string: (“pesticide” OR “organophosphate” OR “organochloride” OR “chlorpyrifos” OR “fumigant” OR “pyrethroid” OR “carbamate” OR “organochlorine”) AND (“neurobehavior” OR “cognitive” OR “motor” OR “psychomotor” OR “sensory-motor” OR “psychological” OR “psychosocial” OR “attention” OR “ADHD”) NOT (“Parkinson” OR “Alzheimer” OR “Suicide”(Mesh)) AND (“Humans”(Mesh)). Articles that met the eligibility criteria were also included through references from chosen articles.

### 2.2. Inclusion and Exclusion Criteria

A total of 950 articles were identified through the search terms, from which 131 articles met the following eligibility criteria: (a) original article; (b) published between May 2009 and September 2021; (c) written in English, Spanish, Italian, French, Portuguese, Russian; (d) evaluating long-term or chronic exposure to low, medium, or high levels of insecticides not reaching the threshold for acute poisoning; (e) using specific tests or questionnaires to assess changes in neurobehavioral aspects or relying on official diagnosis from a physician, neurologist, or psychiatry. After reading the content of the articles, we observed that there are publications that address the same study cohorts with the same biological samples (urine, cord blood from pregnant women or children) for exposure assessment and evaluation of the neurobehavioral effects at different timelines as the cohort ages. Thus, all the publications on the same cohort were combined, and their results are given as one cohort study result. In the final analysis, 86 study results have been included.

As this systematic review was specifically on the neurobehavioral effects of long-term insecticide exposure, all the articles with Parkinson’s Disease, Dementia, and Alzheimer’s disease were excluded. Case reports of single cases of acute poisoning requiring hospitalization were not considered eligible for the review.

### 2.3. Data Analysis

Studies that met the inclusion criteria were sorted into categories based on compounds (organophosphates and/or carbamates, pyrethroids, multiple or undefined insecticides, organochlorines) and based on exposure type (occupational, non-occupational exposure). The non-occupational exposure studies were divided into 2 groups, depending on the residency area of the study population: “rural area residents neighboring agricultural fields” and “other populations”. The studies on rural area residents predominantly compared the target population with residents of nearby cities or towns and/or calculated the proximity of houses from the insecticide-applying agricultural fields and/or measured involvement of the target population in fieldwork and/or included in exposure measurement frequency of agricultural insecticide use in the neighborhood area. The general population study group concentrated on the frequency of indoor/outdoor insecticide use and dietary intake of insecticide residues (frequency of fruit, vegetable consumption, soaking time, washing before eating habits, etc.).

The articles were scored and ranked according to the following criteria: (a) study design; (b) sample size; (c) target population; (d) control group; (e) exposure assessment; (f) neurobehavioral domain assessed; (g) neurobehavioral tests or questionnaires used; (h) exposure-effect estimate; (i) control for confounders; (j) reported conflict of interest. For this review, 7 parameters were used to give a score for the methodological quality of each study. Each study was assigned a score of 0 to 2–3 for each of the 7 parameters. The studies were classified as follows: Tier 4 category (0–10 out of the 18 points), Tier 3 (11–13 points), Tier 2 (14–15), and Tier 1 (16–18 points). The studies rated in Tier 1 and Tier 2 categories were given more consideration in our conclusions than those in Tier 4 and Tier 3 categories. The ranking system was based on a similar system used in the review by Muñoz-Quezada [18] for the same purposes. In Table 2 is illustrated the scoring system, with a detailed description of the requirements. The sample size for the occupational exposure studies is scored for a different number of participants considering the difficulties in recruiting agricultural workers for the studies due to the smaller pool compared to the other population.

The main neurobehavioral effects observed in the studies were arranged into four domains: (1) cognitive function (CF), which includes intellectual quotient (IQ), mental development, memory, language, and reasoning; (2) psychomotor function (PM), which includes motor skills; (3) sensory-motor function (SM), which includes auditory, visual, and olfactory stimulation; and (4) psychosocial function (PF) or behavioral, which includes adaptive behavior, inhibitory control, social development, depression, anxiety, and mood disorders. In studies on possible prenatal exposure, the PF domain also included attention deficit hyperactivity disorder (ADHD) and autism spectrum disorders (ASD). The summary of the methods used for the evaluation of the possible changes in neurobehavioral functions of the population, categorized by the exposed group, the areas and the frequency of appearance is given as supplementary materials (Table S1).

## 3. Results

Out of the 950 eligible scientific articles identified, 86 studies were selected for the final review process. Twenty-six of them were on occupational and 60 on non-occupational exposures. Among the latter group of studies, 17 addressed rural area residents and 43 the general population.

The insecticides most frequently addressed were organophosphates, with 24 on non-occupational and 12 on occupational exposures. In 23 studies, pesticide compounds were not identified but referred to as “insecticides” in general. Exposure to pyrethroids was assessed in 17 studies, 10 of them for the general population. Exposure to organochlorine compounds was estimated in 21 studies, and only for non-occupational exposure. Other insecticide compounds mentioned in the studies were mancozeb [19], fumigants [20], and carbamates [21,22].

There were 12 studies on non-occupational exposure and two studies on workers’ exposure in the Tier 1 category. Studies in this category were generally national big cohort studies with a follow-up time of over 1 year and a sample size of over 300, with a complex pesticide exposure assessment and several neurobehavioral tests applied to participants. In particular, we found 19 non-occupational and 7 occupational studies in the Tier 2 category. These studies were generally big cross-sectional studies (over 1000 study subjects) with data from nationwide surveys or cohorts. Tier 3 category studies were usually small sample size longitudinal studies with questionnaires as exposure assessment methods or cross-sectional studies with the research with a questionnaire of self-reported neurobehavioral changes. The studies within the Tier 4 category are cross-sectional studies characterized by very small sample size (less than 50), with experimental tests used for the assessment of neurobehavioral changes in a target population. In these studies, the risk of bias is very relevant due to incomplete statistical analysis of the data, lack of control for confounders, or unclear exposure assessment methods. A summary of the quality assessment of studies included in this review is presented in Table 3.

**Figure 1:**
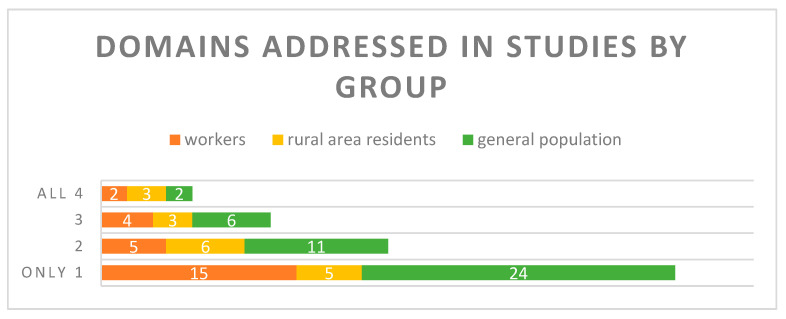
Domains addressed in studies by group.

Concerning the neurobehavioral tests, 51% of all the studies included in our review took into consideration only one neurobehavioral domain, usually cognitive or psychosocial, ignoring other domains (Figure 1). Only seven studies out of eighty-six used the whole set of neurobehavioral battery tests to assess all four domains, and only one study on occupational exposure used, for the assessment of neurobehavioral effects, the WHO recommended neurobehavioral core test battery. In the studies evaluating two domains, a combination of cognitive and psychomotor or cognitive and psychosocial domains was the most popular. Among the studies appraising only one neurobehavioral domain, psychosocial and cognitive functions were the most prevailing interest for the authors. The psychosocial functions were predominantly tested in case-control studies with children with ADHD or ASD, while the cognitive function was mostly tested for the elderly, adult population and children.

### 3.1. Occupational Exposure

Occupational exposure studies were mostly conducted on farmers, pesticide applicators, and sheep dippers. The biggest cohort studies in this group were from industrialized countries, which are also the ones that often have stringent safety rules, where the levels of exposure are usually lower and obsolete insecticides are not used. Developing countries, where the need for these studies is probably higher, have been addressed only by a few small studies. The USA is the most represented country, based on the number of studies conducted there.

Generally, in the studies dealing with occupational exposure, the most common study plan was cross-sectional, based on the comparison of exposed versus unexposed controls. Usually, the control groups were subjects living in the same area as the exposed groups. The levels of education were often different (higher in the control group), and this difference must be considered as a possible source of bias. In some studies, different levels of exposure have also been considered, as well as the presence, in the anamnesis, of a previous severe insecticide poisoning. Almost all the studies collected information regarding previous insecticide poisonings, including the related severity. In the longitudinal studies, estimates of cumulative exposure were carried out with different approaches, depending on the data available. In addition, Geographic Information Systems (GIS) data and data from the local register of insecticide use were used in a few studies, but not in all of them.

In some studies, the levels of insecticide exposure were assessed through the measure of biological indicators of dose, which is blood or urine concentration of specific metabolites, or through the determination of early biochemical effects, such as acetylcholinesterase (AChE) and butyrylcholinesterase (BChE) inhibition levels for organophosphates and carbamates exposures. In other studies, the exposure levels were estimated through questionnaires or by using, as a proxy of exposure, the employment in a farm of the study subjects, or their residence in a rural area, without clarifying the frequency of contact with specific insecticides.

When the ranking system was applied to occupational exposure studies, only PHYTONER [12,23] and Anger et al. [24] studies scored as Tier 1. Seven studies were given rank as Tier 2 [16,25,26,27,28,29,30,31], while seventeen studies were ranked as Tier 3 or Tier 4.

Studies with a longitudinal design had only one follow-up, usually with the baseline before insecticide application season and the follow-up after it. Sixteen out of twenty-six studies were cross-sectional; however, among Tier 1 and Tier 2 classes, only three out of nine were cross-sectional, with a sample size of over 200 participants. The question of the correlation between depression and anxiety in farmers and the level of exposure to insecticides in general among studies with Tier 2 ranking have been evaluated by Koh et al., Beard et al., Weisskopf et al., and Kori et al. [28,29,30,31].

The series of longitudinal studies on Egyptian adolescent insecticide applicators [25,26,27,32] assessed at a different time all the behavioral domains. Fifteen studies presented results for one domain only (seven of them on depression and anxiety, six on sensory-motor functions and two on cognitive impairment). Among them, three studies investigated the effects of exposure to insecticides on auditory functions, one on olfactory functions and one on postural control. The cognitive domain was investigated in 13 studies, where MMSE and WAIS were the most frequently used tests. Four studies reported the possible effect of occupational insecticide exposure on memory and attention, although none of them could affirm a clear correlation between metabolite concentrations or lifetime insecticide exposure indexes and lower scores on tests. Most of the neurobehavioral outcomes were correlated to the occupation of the participant in comparison with the control group. Four studies out of thirteen included participants with past insecticide poisoning. Subjects in three other studies reported the absence of PPE use during insecticide application. Overall, though the trend of lower scoring in the cognitive domain of farm workers is clear, the influence of higher exposure levels due to the absence of PPE or the influence of past acute insecticide poisoning on current results cannot be ignored.

The PHYTONER study pointed out a reduction in motor speed/coordination, correlated with exposure to OPs, Similar results were observed in a study of sheep dippers carried out in the UK. Some studies suggest that the auditory functions of farmers might have been affected by their exposure to insecticides, while other studies report limited evidence for it. Each of the studies on sensory-motor domain functions used different neurological tests, some of which were experimental, and neither of them was able to maintain an adequate insecticide exposure measurement. All occupational studies on psychological functions investigated the effects of insecticide exposure on depression and anxiety. While 11 of the 14 studies report positive results, it should be noted that participants in all these studies had higher-than-usual insecticide exposure due to past insecticide poisonings and limited use of personal protective equipment (PPE).

Interestingly, in the subjects with a previous episode of acute insecticide poisoning, the neurobehavioral changes were more evident than in the subjects without such an event in their personal history. In addition, the non-use or improper use of PPE tended to be correlated with poorer performance on neurobehavioral tests.

### 3.2. Rural Area Residents

The studies on exposure to insecticides in rural area residents were generally characterized by complex exposure assessment methods. In most of the studies, rural area residents were evaluated according to the proximity of their houses to the fields or greenhouses, the frequency of insecticide use in these settlements, or their involvement in farms. The rural area residents were usually compared to the nearby town population or stratified depending on their house proximity from the field and/or occupational exposure of the parents and/or the levels of metabolite concentration in blood, urine, or breast milk. Almost all the studies were on children living nearby agricultural fields or children prenatally exposed to insecticides due to their parent’s occupation.

Five out of seventeen studies were ranked Tier 1 in this group. Most of the studies (9/17) on rural area residents assessed the exposure to organophosphates, whilst six studies were on exposure to multiple insecticides. Few studies concentrated on organochlorines and pyrethroids, and some mentioned mancozeb, carbamates, and fumigants. Nine studies were longitudinal in design, seven were cross-sectional, and one was an ambispective study. The number of participants varied from 50 to 400. In three studies, all four domains of neurobehavioral development were evaluated, in three studies only the cognitive domain was assessed, and six studies on rural area residents were used to evaluate two domains in the neurobehavioral development of children; the other studies evaluated only one neurobehavioral domain. The most frequently used tools were WISC-IV and BARS.

The results are not consistent between the studies. For example, the CHAMACOS study on pregnant women, which followed children from the neonatal period up to 15 years old, reports that decrements in cognitive functions in children were associated with higher prenatal, but not postnatal, exposure to organophosphates, with residential proximity to agricultural fields and with a higher concentration of DDE in serum. Psychomotor functions were reported as developing normally. Among psychological functions, authors correlated exposure with ADHD, but not with ASD. On the other hand, another study reports motor development decrements in children with exposure to propoxur, but not cognitive or psychological function impairments [33]. In addition, a reduction in neurobehavioral performance related to pyrethroid exposure [34], while the study by Ostrea et al. 2012 [33] reports no association in the same domains with pyrethroids.

One study addressed the elderly and evaluated ambient exposure to OP insecticides from commercial agricultural insecticide applications in proximity to each participant’s residence 5 years before the survey. The study reported that those living in proximity to high agricultural OP application experienced a faster cognitive decline over time on the 3MSE, though out of 41 dementia/CNID cases, only six were from the group with high OP exposure.

Overall, six studies reported a positive correlation between exposure to organophosphates and decrements in the cognitive domain; four of these studies were ranked either Tier 1 or Tier 2. Among cognitive functions, three studies report poorer performance on attention, memory, and Full-Scale IQ. Two studies report significantly poorer performances in boys, but not in girls. Among studies with mixed insecticide exposure, only four investigated the cognitive domain, two of them had limited evidence, and two had negative results.

### 3.3. General Population Exposure

We included in this group studies addressing the general population, whose exposure to insecticides was limited to the use of indoor or outdoor insecticides and/or dietary intake, such as studies based on the data from the NHANES surveys or any other study addressing exposure to insecticides of the general population. In this group, the largest among the three addressed, we found 18 studies on exposure to organochlorines, 15 on organophosphates and 10 on pyrethroids, for a total of 43 studies. Seven studies addressed multiple insecticide exposure without any specification of single compounds. Seven of these studies were ranked Tier 1 and sixteen Tier 2. Out of 43 studies, 23 were on prenatal exposure, 17 were on postnatal exposure, and 3 were on the elderly. The studies on prenatal exposure usually had a longitudinal design, while the studies on children were mostly cross-sectional. For exposure assessment, all the studies used metabolites’ concentration in bodily fluids as indicators of dose. In some of these studies, the exposure assessment was completed by questionnaires.

Among the studies taken into consideration, all four neurobehavioral domains were evaluated in two studies only. In twenty-four studies, only one domain was evaluated; in particular, thirteen studies were on the psychosocial domain, six on cognitive functions, and five on motor functions. Cognitive and psychomotor functions were evaluated together in six studies. Fourteen studies were on attention deficit hyperactivity and autism spectrum disorders. Neurobehavioral effect assessment tools varied broadly: Bayley-II, WISC-IV, WPPSI, and BASC were used for bigger longitudinal studies with big cohorts, while SRS and SDQ were used for the psychosocial domain.

Among the studies addressed to organophosphates, two reported a positive association with a reduction in performance on cognitive functions, whilst two did not report any association and one reported only limited evidence. Two studies reported lower scores in working memory index (WMI) and mental development index (MDI) in children associated with higher Dialkylphosphate (DAP) metabolite concentrations in mothers’ urine during pregnancy. In one of the biggest cohorts of prenatal exposure [35], MDI was higher in white women’s children, despite their high levels of prenatal ƩDAP (the sum of dimethylphosphate, dimethylthiophosphate, diethylphosphate, and diethylthiophosphate) concentrations. This could be explained by this subgroup’s higher rate of consumption of fruits and vegetables, which may contain preformed DAP residues. The authors of this study also report higher executive functions in black people, presumably due to the small number of participants and, as a result, broad confidence intervals in the results.

In the only study investigating the presence of tremors, prenatal exposure to chlorpyrifos (CPF) was associated with this symptom in nine-year-old children in a cohort from New York, NY, USA [36]. Another study [37] reported worse motor development in girls and lower visual acuity associated with CPF exposure, whilst the other three studies ranked Tier 1 and Tier 2 failed to show any association between motor development and OPs’ exposure.

Among psychosocial studies, three out of seven reported a positive association between poorer social behavior and OP exposure, two reported a negative association, and two reported only limited evidence. Three studies reported a positive association between exposure to pyrethroids and organochlorines with behavioral changes, in particular with attention-related problems. Orenstein et al. [38] found a positive association between ADHD in children and organochlorine exposure in mothers during pregnancy, although the study was on a cohort from the New Bedford Harbor Superfund Site highly contaminated with PCBs from industrial waste from the 1940s through to 1977. Xu et al. [39] pointed out an association between postnatal 2,4,5-trichlorophenol (2,4,5-TCP) and 2,4,6-trichlorophenol (2,4,6-TCP) levels in urine and ADHD in US children (Red). However, the evidence of an association between ADHD and OPs exposure is questionable; in fact, only Bouchard et al. [40] reported a positive association between DAP metabolite concentrations in children and ADHD diagnosis, while in the ELEMENT cohort, chlorpyrifos metabolite—3,5,6-trichloro-2-pyridinol (TCPy) levels in the highest tertiles were associated with ADHD in boys only.

Autism-related disorders were reported to be associated with OP exposure in one study on a cohort of mothers [41], who already had a child with autism. The association was positive for girls, but not boys with a higher concentration of urinal dimethyl thiophosphate. The Mount Sinai cohort reported a positive association with a higher score on The Social Responsiveness Scale (SRS), whose results are associated with ASD, ADHD, and other neuropsychiatric conditions, with exposure to OPs. Considering other insecticide compounds, Lyall K et al. [42] reported no association between organochlorine compounds and ASD, while Schmidt et al. [43] found that prenatal exposure to insecticides, in general, might be correlated with ASD in children.

## 4. Discussion

### 4.1. General Considerations

To our knowledge, this is the first attempt to systematize the studies on insecticide exposure in both occupational and general populations. While the workers are for sure the most highly exposed group to agricultural insecticides, there are an increasing amount of cohort studies on environmental exposure among the vulnerable population, especially pregnant women and children. Considering that occupational exposures to insecticides are greater than those of the general population, we have decided to discuss them separately.

Though many insecticides have neurotoxic effects, most of the available studies address OP exposure. In several studies on organophosphates, exposure was assessed through the determination of DAP, OP’s metabolite, in urine, but these measures have a high risk of providing incorrect estimates of real exposure because DAPs can be both produced in humans by OP’s metabolism, and also in the environment by abiotic hydrolysis, photolysis, or plant metabolism. OP’s metabolites have a short half-life, and because of this they cannot be a good marker of the whole past exposure, but only a photograph of the actual point in time. Because DAP is not neurotoxic [44,45,46], for subjects whose primary source of insecticide exposure was fresh fruit and vegetable consumption, the use of urinary metabolite concentrations as an indication of parent compound exposure may result in significant misclassification of exposure [35]. Chronic exposure to organochlorines was usually assessed together with persistent organic pollutants on cross-sectional studies and mixed pesticides, but the best way to assess the cumulative exposure is by using a retrospective exposure assessment. Studies on pyrethroids had a common limitation of low detection levels in the population.

Several studies aimed to investigate the general health effects of environmental exposure, including insecticides, with follow-ups over decades to assess the long-term prenatal and postnatal exposure. The common limitation of these longitudinal studies was a high percentage of dropouts from the primary cohort, very high LODs for detection of insecticide metabolites, subgroups of participants with exposure levels labeled as “below LOD”, and incomplete data from participants. The main limitation of the available studies was that only 8% of them investigated the whole spectrum of neurobehavioral functions, whilst half of them addressed only one neurobehavioral function domain, ignoring the others, which might lead to some lapses in the final results, demonstrating only the “tip of the iceberg”. A list of the tests used in the papers is shown in the supplementary materials (Table S1). For example, it is known that perception and cognition guide movement and action as much as action influences perception and cognition [47]. Leonard [48] confirms that motor development not only provides opportunities for the development of a range of perceptual, social, and cognitive skills, but is also influenced in turn by these abilities in an interactive process. Therefore, evaluating the neurotoxicity of the compound through only one neurobehavioral domain might lead to a “patched” view of the situation, hiding the real size of the problem. Moreover, the methodological approaches used in the studies were often widely diversified. The results obtained by various neurobehavioral tests were difficult to compare and very seldom the hypothesis generated by single studies found a firm confirmation by another study, hence no firm conclusion can be drawn about the impact of insecticide exposure on neurobehavioral functions.

### 4.2. Occupational Exposure

The profile of agricultural workers’ occupational exposure to insecticides may change depending on the country’s policies, but any agricultural worker might be exposed to insecticides. The youngest participants with high direct exposure were nine-year-old Egyptian seasonal workers applying insecticides to cotton fields [49]. This series of studies on children and adolescents was the only one addressing the problem of younger population exposure in agricultural workers [25,26,27,32,50,51,52]. A dose-dependent reduction in neurobehavioral functions was observed in this cohort, in association with the duration of exposure. Another study, including working adolescents from Iran, also reported a positive relationship between exposure levels and poorer performance on neurobehavioral tests [53]. Elderly people (>65 years old) are another vulnerable worker subgroup, despite being included commonly in the adult workers cohort [54].

One more subgroup of workers investigated in the studies was the workers who reported “unusually high insecticide exposure events”. The Agricultural Health Study [55] cohort reported past insecticide poisoning symptoms and signs without hospitalization. These were cohorts with “small or no use of PPE” from developing countries. This subgroup of agricultural workers has been reported to have poorer performance on cognitive functions compared to non-exposed or less exposed. Most of the studies addressing occupational exposure did not carry out any exposure measurement, and in some of them the type of exposure was also not reported. In the studies in which the levels of exposure have been measured through the determination of biological indicators of dose, in some cases in combination with a questionnaire, a clear dose-response relationship was observed only in a study on children and adolescents [49], but because OP’s metabolites have a short half-life, they cannot be a good marker of the entire past exposure, but only a snapshot of the current point in time. Interestingly, almost all the studies included a question about previous insecticide poisoning in their questionnaires. Most of the studies included general insecticide poisoning only, without other details, but many studies reported poorer performance on neurobehavioral domains associated with the history of past insecticide poisoning. In these cases, the poor performance could be due to a generic anoxic injury, as observed, for example, in the Sarin-poisoned subjects in the Tokyo Underground terroristic attack [56], in subjects previously poisoned by carbon monoxide [57] and even as a consequence of severe head trauma [58]. However, the PHYTONER study [12,23], ranked as Tier 1, was the first one to provide prospective data on the natural history of neurological disorders associated with insecticide exposure and reported a higher risk of dementia in workers with direct exposure to insecticides, even excluding the participants with past acute poisoning. Other studies in Tier 2 reported a negative association between decrements in the cognitive domain and exposure without past acute insecticide poisoning [59], while the previous acute poisoning was strongly associated with depression and anxiety in workers [21,28,29] and with a decrease in the cognitive domain [21].

Studies on psychomotor functions of the workers reported that lower scores on finger tapping and motor speed/coordination were positively correlated with exposure to OPs, although the same dependence from past insecticide poisonings or “high” exposure levels can be observed in participants. The sensory-motor domain has been investigated in several Tier 3 and 4 studies, often using experimental methods to assess neurobehavioral effects; nevertheless, none of them maintained an adequate insecticide exposure measurement or provide comparable results to draw a univocal conclusion.

Valid assessment of the mood and emotional status of the workers is difficult due to its subjectiveness, quantification problems, and hard-to-rule-out confounding factors. The main limitation of these studies is the fact that they addressed only the psychosocial domain and therefore a comprehensive view of neurobehavioral functions is absent.

### 4.3. Non-Occupational Exposure

Rural area residents have higher levels of exposure to insecticides due to their proximity to agricultural fields and involvement in fieldwork compared to the urban or suburban area population. Almost all the studies in this group were on children, living nearby agricultural fields, or children prenatally exposed to insecticides due to their parent’s occupation and/or close proximity to large farms.

The residential proximity from the agricultural fields treated with OP compounds during pregnancy was associated in the CHAMACOS study with poorer cognitive functioning in children at 10 years of age [60]. On the other hand, González-Alzaga et al. [61] report that cognitive function decrements in children were more associated with postnatal exposure depending on the distance from the treated field, while prenatal exposure had a weak association. Additionally, residence proximity from the agricultural field applying more OP insecticides was positively associated with faster cognitive decline in elderly Mexican-Americans, as was reported by the SALSA study [62].

Neurodevelopment till five years old was addressed in a few general population studies, where measured MDI, in general, was not associated with organophosphate metabolite concentration levels, neither during pregnancy nor during childhood. However, in rural area children, the CHAMACOS Study reported a decline in MDI in children of two years associated with prenatal exposure. There were only six studies on the effect of organophosphate compounds on the neurobehavioral development of children aged between five and fifteen years old with Tier 1 or Tier 2 ranking, including rural area residents. The Mount Sinai cohort of New Yorkers reported an association between lower WMI, perceptual reasoning, and Full-Scale Intelligent Quotient test (FSIQ) and higher DAP concentrations in mothers during pregnancy. The same effects were observed in the CHAMACOS Cohort on Mexican-Americans from rural areas. The Columbia Center Cohort confirmed decrements in WMI and FSIQ. However, European mother–child cohorts related poorer performance with postnatal exposure rather than prenatal, reporting slight decrements in WMI scores (The PELAGIE Cohort) and Processing Speed and FSIQ (Spanish Cohort). Contradictorily, the HOME Study cohort from Ohio reports a negative association with any of these cognitive functions and exposure to OPs.

The psychomotor development index has been reported to have no associations with exposure to organophosphates in infants and toddlers; however, Silver et al. [37], using the Peabody Developmental Motor Scales 2nd edition (PDMS-2), reported that girls at 9 months had decrements in motor development, while Woskie et al. [63], using the Bayley Scales of Infant and Toddler Development-III (Bayley-III), also report some decrements in motor development and visual acuity in five-month-old infants associated with prenatal exposure to chlorpyrifos. On the psychomotor domain of children aged between five and fifteen years old, only the Columbia Center Cohort [36] reported tremors measured by Archimedes spiral test to be correlated with a high concentration of chlorpyrifos in prenatal umbilical cord blood, but other studies [35,40,64] reported no association of exposure to insecticides with any motor function decrements at this age. In the CHAMACOS study, authors report no consistent associations between OP exposure in mothers and children, as measured by urinary DAPs, and children’s autonomous nervous system (HR, RSA, and PEP) regulation during resting and challenging conditions up to the age of 5 years.

According to the reports of the CHAMACOS cohort study, prenatal and, to a lesser extent, postnatal OP exposure was associated with problems with attention and ADHD. However, the association with traits related to developmental disorders such as ASD was unclear. The Mount Sinai cohort study reported an association between increasing prenatal ΣDEP metabolite concentrations and adverse social-reciprocal behavioral deficits among black people and boys, but not with other OP metabolites. In addition, the ELEMENT cohort reports that TCPy levels in the highest tertile were associated with ADHD in boys, but not in girls, although attention problems were more associated with girls. These results are in line with the reports from studies outside of this review, which found that in the clinical presentation of attention deficit disorder, girls display more inattentive-type problems, while boys display more hyperactive and impulsive behaviors, and that generally boys are more likely to be diagnosed with ADHD than girls [65,66,67]. A study on a Chinese cohort reported poorer performance in the social domain associated with prenatal, but not postnatal, exposure [68]. Moreover, the effect of prenatal exposure to OPs might affect the psychosocial domain, affecting the behavior and social interaction skills of children.

Many of the non-occupational exposure studies investigated exposure to both pyrethroids (PYR) and organophosphates in children. Among rural area children, exposure to PYR was not associated with neurobehavioral decrements [33] or was weakly associated with a deficit in language and motor function development in infants of 2 years old [34]. However, other studies on children who live in urban and suburban areas demonstrate different results.

Many organochlorine insecticides were banned from agricultural use several years ago; however, their persistence in nature resulted in accumulation in the human food chain, including mother’s milk [69]. Mother–child cohorts from Japan and Spain reported problems with memory and executive function associated with OCP compounds in mothers’ blood, which was supported by the CHAMACOS cohort study results. However, the cohort from the New Bedford Harbor Site did not demonstrate any association between prenatal OCP exposure and cognitive function scores, though this study was more oriented on mercury exposure. No association was reported by the cohort from South Africa. In addition, no association with poorer performance in the cognitive domain was reported by the cohort from Guadeloupe, although the results might have been affected by the small number of participants. Lyall et al. [42] reported that prenatal exposure to OCPs could be associated with intellectual disability without autism, but not in the highest tertiles of OCP metabolites. No association between motor function impairment and OCP metabolite concentrations was reported in the studies. On the other hand, all the studies addressing ADHD in children reported a positive association with organochlorines, while the association with autism was not observed. Additionally, higher OCP metabolite concentrations in the blood of the elderly have been associated with a faster decline of cognitive functions in Swedish and US cohorts.

In general, the effects of insecticides on the neurobehavioral function of the urban and suburban population were not a popular topic; however, few studies attempted to investigate mixed insecticide effects without determining compounds. The INMA project from Spain [70] was based on the response to a question about the frequency of indoor use of insecticides and reported that prenatal use of spray insecticides was associated with motor function impairment, but it did not affect the cognitive domain.

## 5. Conclusions

Compared to our previous reviews, it is clear that the interest of researchers mainly switched from occupational to environmental exposures. The main focus is still on OP compounds and, to some extent, OCs. Because these compounds have been mostly withdrawn from the market and occupational exposure does not exist anymore, interest has necessarily been focused on environmental exposures to these persistent organic pollutants.

Overall, during the 10 years elapsed since our last literature review, pending doubts have not yet been clarified. In particular, it seems that there is evidence of effects in subjects with a previous severe acute poisoning in their personal history, but this may be a non-specific effect consequent to anoxia [56,57,58] and not a direct toxic effect of insecticides. However, some effects can be observed in the highest levels of exposure. Because children and adolescents are more vulnerable to the neurotoxic effects of insecticides, these data suggest the need to evaluate this potential problem, which requires more comprehensive studies addressing underaged workers’ exposure. In addition, a specific study conducted on elderly farmers may be relevant to better appreciate the long-term effects of exposure to insecticides.

Occupational exposure to insecticides in agricultural workers with previous insecticide poisoning was more correlated with neurobehavioral changes than exposure without acute poisoning in the past. In addition, the insecticide applicators and farmers without personal protective equipment tended to have poorer performance on neurobehavioral tests. Overall, though the trend of lower scoring in the cognitive domain of farm workers is clear, the question of the influence of higher exposure levels due to the absence of PPE and/or the influence of past acute insecticide poisoning on current results remains unsolved, and further studies are needed. Depression and anxiety among workers were more often correlated with high insecticide exposure or past acute poisoning events. Further studies including psychosocial functions and cognitive and/or psychomotor domain are needed to confirm a firm association with chronic exposure in workers. In the general population, despite a tendency of generally inconsistent results in studies over time, some common cognitive functions have been mentioned as affected by different authors, hence the named lower scores on memory, IQ, and attention of children might be due to the higher exposure levels to some OPs, though the question of whether it is more associated with prenatal (CHAMACOS Study) or postnatal [61] exposure remains unclear. Although the results are not fully consistent, there is enough evidence to suggest that prenatal exposure to OPs, in general, might increase the risk of ADHD in children, but the association with ASD remains unclear. Though it seems that prenatal exposure to pyrethroids might affect the behavioral functions of children, there is not yet enough evidence to confirm it.

The main limitations of the studies were the small number of parameters investigated, a questionable approach to retrospective exposure assessment, several different tests used, the small number of subjects studied, and the risk of bias in the selection of control groups, mainly for the different levels of education, or only in the capacity of dealing with the IT. The fact that most of the published studies are cross-sectional means that the prognostic significance of the small changes observed in some cases remains obscure, and it is impossible to decide whether they are early effects able to evolve into overt disease or only adaptive changes devoid of any tendency of clear pathological evolution. Only longitudinal studies and follow-up of the subjects considered can solve these doubts. Another limitation of occupational exposure studies was that the biggest cohort studies with a sample size of over 500 were from countries with stricter safety demands [16,28,29,30,71,72,73,74], while the countries with a higher risk of exposure to insecticides reported few small sample-sized studies or zero studies. The main limitation in studies investigating pyrethroids was the small number of participants with biomarker values above the LOD being revealed in cohorts, probably due to the rapid metabolism of these compounds in humans; pyrethroids are quickly metabolized with half-lives in the order of hours, meaning that they are not a valid tool to assess exposure history [75].

Because some data suggest that both occupational and environmental exposure to insecticides might affect the neurobehavioral functions of exposed subjects, in particular for high exposure levels and vulnerable subgroups, it is necessary to plan new studies, to be carried out with a special effort to define the cumulative levels of exposure, to focus on specific selected risky compounds and to create groups of a size adequate to point out early and mild changes. The possibility of studying some bio humoral indicators of nervous function and the follow-up over time of subjects previously assessed might help in collecting interesting results. Considering the growing capacity to use geographic information systems (GIS) in scientific research and the wide-spread availability of maps, and meteorological data, depending on the availability of the local registry of insecticide use this method might be a good additional tool for the exposure assessment, in particular for rural area residents.

## Figures and Tables

**Table 1:** Neurobehavioral domains, their functions, and an example of assessment tools.

Neurobehavioral Domain	Functions	Assessment Tools
Cognitive	Alertness Attention Memory Perception Intellectual Quotient Mental development Language Reasoning	Symbol Digit Digit Span Visual retention Trail making
Sensory-motor	Motor coordination Balance Postural Visual acuity Auditory Olfactory	Finger tapping Eye-Hand coordination Postural control test Odor identification AVLT
Psychomotor	Vigilance Reaction time Stability	Santa Ana Pursuit aiming Tapping
Psychological	Mood Social development	Neuropsychological tests Clinical evaluation of symptoms

**Table 2:** The scoring system for the ranking of the articles.

	3	2	1	0
**Study design**	Longitudinal for at least one year, follow-ups over 3 times, exposure before the outcome; series of longitudinal studies on a very similar cohort	Longitudinal for a season, exposure before the outcome, experimental	Case-control, cross-sectional	Case study, ecological, ex0ploratory, descriptive
**Sample size** **(occupational studies)**	201 and more	51–200	10–50	<10
**Sample size** **(non-occupational studies)**	501 and more	201–500	50–200	<50
**Control group choice**	-	Same age, education level, gender, occupation from the same region and study period, randomly picked	Occupation, location, or study period differs, not randomly picked or the exposure measurement for the control group is identified differently.	The number of controls is inadequate or education level, gender, age, or occupation differs significantly, which might affect the outcomes
**Exposure measurement**	Specific biomarkers or combined use of methods	General biomarkers	Questionnaire + residency/GIS/reports on insecticide use	Only one of the following methods: questionnaire, exposure measured without biomarkers, used biomarkers but with no association to effects, partial use of biomarkers
**Measuring of effects**	-	Use of standardized and validated instruments, laboratory tests, scanner, specific tests	Screening tests, interviews, questionnaires, full scales (but not validated)	Sections of full scales (standardized or not), clinical records
**Control of confounders**	-	Control of important confounders (parents’ IQ, education level, diseases, exposure to other neurotoxic agents) and standard variables (statistical models’ analysis)	Control of standard variables with an adequate statistical analysis (i.e., age, sex, occupation)	Neither standard variables nor confounders are considered. Inadequate statistical analysis of confounders was performed
**Reported conflict of interest**	No	-	-	Yes

**Table 3:** Summary of the quality assessment of the studies included in the review.

Rank	Number of Studies on Occupational Exposure	Number of Studies on Non-Occupational Exposure
Tier 1 (16–18)	2	12
Tier 2 (14–15)	7	19
Tier 3 (11–13)	13	22
Tier 4 (≤10)	4	7

## Data Availability

No new data were created or analyzed in this study. Data sharing is not applicable to this article.

## Supplementary Materials

The following supporting information can be downloaded at: https://www.mdpi.com/article/10.3390/toxics11020192/s1, Table S1. A summary of neurobehavioral tests and questionnaires used in the selected articles [76,77,78,79,80,81,82,83,84,85,86,87,88,89,90,91,92,93,94,95,96,97,98,99,100,101,102,103,104,105,106,107,108,109,110,111,112,113,114,115,116,117,118,119,120,121,122,123].
