# Supplementary material for: Neurobehavioral Effects of Low-Dose Chronic Exposure to Insecticides: A Review
Source: Toxics. 2023 Feb 19;11(2):192. doi: 10.3390/toxics11020192 (PMC9963921; doi:10.3390/toxics11020192)
Supplement: Supplementary file 1 [file toxics-11-00192-s001.zip › toxics-2208468-supplementary.pdf]

Supplementary Materials

Table S1. A summary of neurobehavioral tests and questionnaires used in the selected articles.

Function	Name of test	Target population (age of exposed)	References *(edition of the test)
Neurobehavioral test sets for at least 3 domains most frequently used in studies			
Verbal Comprehension, Perceptual Reasoning, Working Memory, and Processing Speed. Full scale IQ	Wechsler Intelligence Scale for Children, 4th edition (WISC-IV)	Non-occupational (school age children)	van Wendel de Joode B. et al, 2016; Rowe C. et al, 2016; Gunier R. et al, 2018; Gaspar F. et al, 2015; Bouchard M. et al, 2011; Furlong M. et al , 2017; Engel S. et al, 2011; Sagiv S. et al, 2012 (3rd edition); Andersen H. et al, 2014 (Revised edition); Viel J. et al, 2015; Cartier C. et al, 2016; Rauh V. et al, 2015; Horton M. et al, 2012; Gonzalez-Alzaga B. et al, 2015;
Mental development index (MDI), Psychomotor development index (PDI)	Bayley Scales of Infant Development, 2nd edition	Non-occupational (infants)	Eskenazi B. et al, 2010; Engel S. et al, 2011; Watkins D. et al, 2016; Lovasi G. et al, 2011; Horton M. et al, 2012; Woskie S. et al, 2017 (3rd edition); Donauer S. et al, 2016; Yamazaki K. et al, 2017; J. Forns et al, 2012; Llop S. et al 2013 (1st edition); Torres-Sánchez L. et al, 2009; Bahena-Medina et al., 2011; Eskenazi B. et al., 2018; Jusko TA et al, 2012 (1st edition)
Neurodevelopment	Brazelton Neonatal Behavioral Assessment Scale (NBAS), Neonatal Behavioral Neurological Assessment (NBNA)	Non-occupational (neonates, infant)	Woskie S. et al, 2017; Zhang Y et al. 2015; Bahena-Medina et al., 2011
Behavioral problems, cognitive	A Developmental NEuroPSYchological Assessment (NEPSY-II)	Non-occupational (children)	Rowe C. et al, 2016; Suarez-Lopez J et al, 2013; Sagiv S. et al, 2012
Neurodevelopment	Ages and stages questionnaire (parent report)	Non-occupational (infant)	Cordier S et al, 2015;
Attention, response speed, memory, and coordination	Behavioral Assessment and Research System (BARS)	Occupational, non-occupational (children, adolescent; adult)	Ismail A. et al, 2017; Butler-Dawson J et al, 2016; Fiedler N. et al, 2015; Ismail AA et al, 2018;
Neurobehavioral function	WHO recommended neurobehavioral core test battery (NCTB)	Occupational (adult)	Cui M et al, 2015
Cognitive function test sets most frequently used in studies			
Cognitive impairment	Mini-Mental State Examination (MMSE)	Occupational, non-occupational (adult, elderly)	Corral S. et al, 2017; Munoz-Quezada M. et al, 2013; Baldi I et al, 2011; Bayrami M. et al, 2012; Berent S et al, 2014; Paul KC et al, 2018; Blanc-Lapierre A. et al, 2012;
Visuospatial construction and visual memory	Rey-Osterrieth Complex Figure Test (ROCF)	Occupational, non-occupational (children, adult)	Corral S. et al, 2017; Munoz-Quezada M. et al, 2016; van Wendel de Joode B. et al, 2016
Intelligence and cognitive ability	Wechsler Adult Intelligence Scale Revised (WAIS-R)	Occupational (adolescent, adult)	Munoz-Quezada M. et al, 2016; Ismail A. et al, 2017; Abdel Rasoul G. et al, 2008;
Visual perception, visual memory	Benton visual retention test (BVRT)	Occupational (adolescent, adult)	Munoz-Quezada M. et al, 2016; Rohlman D. et al, 2014; Baldi I et al, 2011; Blanc-Lapierre A. et al, 2012;
Cognitive function	Digit Symbol Substitution Test (DSST)	Occupational, non-occupational (adult, elderly)	Starks S. et al, 2012; Krieg EF 2013;
Memory and attention	Digit span test (DST)	Occupational, non-occupational (children, adolescent, adult)	Corral S. et al, 2017; Rohlman D. et al, 2014; Harari R. et al, 2010
Cognitive function	McCarthy Scales of Children's Abilities	Non-occupational (children)	Torres-Sánchez L. et al, 2009; Osorio-Valencia E. et al, 2015; Puertas R. et al, 2010
Motor function test sets most frequently used in studies			
Manual motor speed	Finger Tapping Test	Occupational, non-occupational (children, adult)	Andersen H. et al, 2015; Baldi I. et al, 2011; Blanc-Lapierre A. et al, 2012; Starks S. et al, 2012; Harari R. et al, 2010
Psychological function test sets			

Depression	Beck Depression Inventory-Second Edition (BDI-II)	Occupational (adult)	Conti CL et al, 2018; Harrison V., MacKenzie Ross S. 2016
Behavioral problems	Conner's Parent Rating Scale-Revised (CPRS-R)	Occupational, non-occupational (children, adolescents)	van Wendel de Joode et al, 2016; Rohlman D. et al, 2019;
Attention, impulse control	Conners' Continuous Performance Test II	Occupational, non-occupational (children, adult)	Andersen H. et al, 2015; Forns J et al, 2012; Sagiv S. et al, 2012; Starks S. et al, 2012; Harari R. et al, 2010; Rowe C. et al, 2016
Neurobehavioral problems	Self-report or parent report based on specific questions	Occupational, non-occupational (children, adult)	Malekirad A. et al, 2013; Rohitrattana J. et al, 2014; Ismail AA et al, 2017;
Behavioral problems	Child Behavior Checklist (CBCL)	Non-occupational (children)	Rowe C. et al, 2016; Eskenazi B. et al, 2010;
Behavioral problems	Behavior Assessment System for Children (BASC), all versions	Non-occupational (children)	Gunier R. et al, 2018; Sagiv S. et al, 2018; Furlong M. et al , 2017;
Behavioral problems	Strengths and Difficulties Questionnaire (SDQ)	Non-occupational (children)	Viel J-F. et al, 2017; Oulhote Y, Bouchard MF. 2013
Behavioral problems (Autism spectrum and related disorders)	Social Responsiveness Scale, Version (SRS-2)	Non-occupational (children)	Sagiv S. et al, 2018; Furlong M. et al, 2014; Millenson ME et al, 2017
Attention deficit/hyperactivity disorders	The Diagnostic Interview Schedule for Children (DISC-IV)	Non-occupational (children)	Bouchard M. et al, 2011; Wagner-Schuman M. et al, 2015
ADHD, ASD, depression, dementia/cognitive impairment	Clinically diagnosed case-control studies and/or questions such as: "Has the doctor ever diagnosed you/child with…?"	Non-occupational, occupational (children, adult, elderly)	Yu C. et al, 2016; Philippat C. et al, 2018; Beseler C. et al, 2008; Beard J. et al, 2014; Lee D-H et al, 2016; Weisskopf MG et al, 2013; Xu X et al, 2011; Christian MA et al, 2018; Lyall K. et al, 2017; Schmidt R. et al, 2017; Quirós-Alcalá L. et al, 2012; Domingues VF et al, 2016
Test sets used only in 1 or 2 studies			
Cognitive function	Stroop Test; d2 Test of Attention; Frontal Assessment Battery (FAB); Semantic Verbal Fluency, Barcelona Sub-test for Animals and Letter P; The Five Words Test (FWT), Part A of the Trail Making Test (TMT), The Isaacs Set Test (IST),<br>Grooved Pegboard<br>Wide Range Achievement Test-Third Edition (WRAT-3); Reaction time (RTI); Rapid Visual Information Processing (RVIP); Matching to Sample Visual Search Change Score in milliseconds (MTS); Delayed Matching to Sample (DMS); CogniSyst Story Recall Test (CSRT); Stockings of Cambridge (SOC) Moves; Motor Performance Series (MPS); Brief Symptom Inventory (BSI)<br>Event-related potential (ERP)<br>Spanish English Neuropsychological Assessment Scales (SENAS), Spanish and English Verbal Learning Test (SEVLT)	Occupational (adult)<br>Non-occupational (elderly)	Corral S. et al, 2017; Baldi I. et al, 2010; Blanc-Lapierre A. et al, 2012;<br>Starks S. et al, 2012<br>Berent S. et al, 2014<br>Dassanayake T. et al, 2009<br>Paul KC et al, 2018
Motor function	Auditory Verbal Learning Test (AVLT), Sequences	Occupational (adults)	Starks S. et al, 2012<br>Sunwook K. et al, 2016

	Otoscopy, audiometry, random gap detection test, dichotic digit test<br><br>Postural control test<br><br>Transient Evoked Otoacoustic Emissions (TEOAE) and Distortion Product Otoacoustic Emissions (DPOAE)<br><br>Visual evoked potentials<br><br>Peabody Developmental Motor Scales (PDMS-2)<br><br>Eye-Hand Coordination subtest of the Frostig Developmental Test of Visual Perception, 2nd edition (DTVP-2)<br><br>Wide Range Assessment of Visual Motor Ability (WRAVMA) pegboard test<br><br>Snellen chart, Lanthony Desaturated D-15 (LDD-15)<br><br>Teller acuity card (TAC), Auditory brainstem response (ABR)<br><br>Odor identification; odor threshold;<br><br>Fagan test of infant intelligence (FTII),<br><br>Brunet-Lezine scale of psychomotor development of early childhood (revised Brunet-Lezine)	Non occupational (children)	Alcarás PAS et al, 2013;<br><br>Cartier C. et al, 2014<br><br>Silver M. et al, 2017<br><br>van Wendel de Joode B. et al, 2016;<br><br>Sturza J. et al, 2016<br><br>Quandt SA et al, 2017<br><br>Dallaire R. et al, 2012
Cognitive function or neurodevelopment	Serial digit learning, Simple reaction time, Stanford–Binet IV Memory for Sentences; The Woodcock–Johnson III Tests of Cognitive Abilities (WJ-III)<br><br>Verbal Comprehension; Raven's Colored Progressive Matrices; Archimedes spiral<br><br>Chinese Binet test, arithmetic test, picture completion test, maze test, and cancellation test<br><br>Gesell Developmental Schedules (GDS)<br><br>NICU Network Neurobehavioral Scale (NNNS)<br><br>Mullen Scales of Early Learning; MacArthur–Bates Communicative Development Indices<br><br>the Developmental Coordination Disorder Questionnaire<br><br>2007 (DCDQ'07). Parent report<br><br>Cultural fair intelligence tests (Cattell 2 test)<br><br>Graham–Rosenblith Scale<br><br>Wide Range Assessment of Memory and Learning (WRAML)<br><br>Wechsler Preschool and Primary Scales of Intelligence-III (WPPSI-III)	Non-occupational (children)	Andersen H. et al, 2015; Krieg EF 2013;<br><br>Rauh V. et al, 2015<br><br>Wang N. et al, 2016<br><br>Wang Y. et al, 2017; Guodong D. et al, 2012<br><br>Yolton et al, 2013<br><br>Pan I-J et al, 2009<br><br>Høyer et al., 2015<br><br>Hallit S. et al, 2019<br><br>Bahena-Medina et al., 2011<br><br>Orenstein S. et al, 2014;<br><br>Furlong M. et al , 2017; Engel S. et al, 2011<br><br>Ostrea EM et al, 2012

	Griffiths Mental Developmental Scale		
ASD<br><br>Depression<br><br>Behavioral problems	ADOS (Autism Diagnostic Observation Schedule), Social Communication Questionnaire (SCQ), Mullen Scales of Early Learning (MSEL)<br>[Brief Symptom Inventory (BSI), Postpartum Bonding Questionnaire (PBQ), Mother-to-Infant Bonding Scale (MIBS) and Edinburgh Postnatal Depression Scale (EPDS)]<br>Hospital Anxiety and Depression Scale<br>Beck Anxiety (BAI)<br>Short-Form Geriatric Depression Scale (SGDS)<br>Hillside Behavior Rating Scale<br>Behavior Rating Inventory of Executive Functioning (BRIEF)<br>Facial Expression Recognition Test	Non-occupational (children, mothers)	Philippat C. et al, 2018<br><br>Yalçın S. et al, 2014<br><br>Harrison V., MacKenzie Ross S. 2016<br><br>Koh S-B. et al, 2017; Kim J et al, 2013<br>Rowe C. et al, 2016<br>Furlong M. et al, 2017<br><br>Sagiv S. et al, 2018
